# Effects of ultrasound-guided nerve block combined with PCIA analgesia on postoperative pain, inflammatory response, hospital stay, and adverse reactions in breast cancer surgery

**DOI:** 10.4314/ahs.v25i1.24

**Published:** 2025-03

**Authors:** Xiaojuan Zhou, Mingkai Yu, Xiaojing Xie

**Affiliations:** 1 Department of Anesthesiology, Northwest Women and Children's Hospital, Xian, China; 2 Department of Anesthesiology, Baoji Hospital of Traditional Chinese Medicine, Baoji, China; 3 Department of Anesthesiology, Baoji People's Hospital, Baoji, China

**Keywords:** Ultrasound-guided, thoracic nerve block, patient-controlled intravenous analgesia, breast cancer, inflammatory factors, adverse reactions

## Abstract

**Background:**

This study aimed to evaluate the effects of ultrasound-guided combined nerve block with patient-controlled intravenous analgesia (PCIA) on postoperative pain, inflammatory response, hospital stay, and adverse reactions in breast cancer surgery.

**Methodology:**

A total of 100 patients undergoing radical mastectomy for breast cancer were randomly assigned to two groups. The combined group received ultrasound-guided thoracic nerve block with PCIA, while the PCIA group received PCIA alone. Visual Analogue Scale (VAS) scores for pain were assessed postoperatively at intervals, and levels of inflammatory response markers (IL-6, TNF-α, hs-CRP) were compared before and after surgery. Analgesic efficacy, sufentanil dosage, hospitalization conditions, and safety profiles were recorded.

**Results:**

The combined group exhibited significantly lower VAS scores for resting and active pain at various postoperative time points compared to the PCIA group. Inflammatory markers at 48h post-surgery were notably lower in the combined group. Sufentanil consumption, analgesia pump use, rescue analgesia rate, and duration of effective pain relief were all improved in the combined group (P<0.05).

**Conclusions:**

Ultrasound-guided nerve block combined with PCIA effectively reduced analgesia pump usage, alleviated postoperative pain, and suppressed inflammatory marker expression. However, it showed minimal impact on hospital stay and adverse reactions, making it a promising strategy for postoperative pain management in breast cancer surgery.

## Introduction

Breast cancer, the most common malignancy in women, is typically treated with radical mastectomy, which can lead to significant postoperative pain and stress, hindering recovery^1^. Patient-controlled intravenous analgesia (PCIA) is a commonly used postoperative analgesic method in clinical practice, but the use of narcotic analgesic drugs in excessive amounts may cause immunosuppression, drowsiness, gastrointestinal discomfort, respiratory depression and other adverse reactions, which may adversely affect the postoperative recovery process. Recent studies highlight the evolving understanding of postoperative pain management in breast cancer surgery, underscoring the need for innovative approaches that minimize opioid use and enhance recovery^2^,^3^. These advancements set the stage for our investigation into ultrasound-guided nerve block combined with PCIA, a technique poised to address current challenges in pain management. Nerve block is an important part of multimodal analgesia, which has a certain role in reducing the number of perioperative opioids and reducing the degree of postoperative pain, and the application of ultrasound guidance can make the nerve block operation more accurate and provide good analgesic effect. However, the duration of the analgesic effect of nerve block is relatively short, so it is often used as an auxiliary analgesic method in combination with drug analgesia^4^. The aim of this study was to investigate the effect of ultrasound-guied nerve block combined with patient-controlled intravenous analgesia (PCIA) compared with PCIA alone in postoperative pain management of breast cancer, with a view to providing a more effective pain control strategy for clinical use.

## Materials and Methods

### Patients

This study consecutively enrolled 100 patients who underwent radical mastectomy for breast cancer in our hospital from January 2021 to August 2023, and the above patients were divided into two groups by simple randomization method. This study strictly adhered to ethical guidelines, and all participating patients were fully informed of the nature, purpose, possible risks and benefits of the study and signed an informed consent form before the study began. In addition, patient data privacy was strictly protected during the study to ensure the safety of all personal information. We meticulously defined our patient selection criteria to ensure the study's reproducibility. Inclusion criteria encompassed adult female patients diagnosed with breast cancer via puncture biopsy, scheduled for radical mastectomy under general anesthesia, with BMI ≤ 26 kg/m^2^. Exclusion criteria included known allergies to anesthesia drugs, coagulation disorders, long-term use of analgesics or corticosteroids, and organ dysfunction. These criteria aimed to create a homogeneous study population while addressing potential confounding factors.

Inclusion criteria: 1) all patients were diagnosed with breast cancer by puncture biopsy^5^; 2) all were adult female patients; 3) all underwent radical mastectomy under general anesthesia; 4) the patients themselves signed the consent form.

Exclusion criteria: 1) allergy to anesthesia drugs; 2) coagulation disorders or endocrine system diseases; 3) history of long-term analgesic drugs or corticosteroids; 4) dysfunction of the heart, liver, kidney and other organs; 5) puncture site ulcerated and infected; 6) body mass index (BMI) >26kg/m^2^. The combined group received ultrasound-guided thoracic nerve block combined with patient-controlled intravenous analgesia (PCIA) intervention, with an age range of 35 to 75 years (mean age 53.96 ± 10.47 years). The PCIA group received PCIA analgesia alone, with an age range of 35 to 75 years (mean age 52.77 ± 11.02 years). The general characteristics of patients undergoing radical mastectomy for breast cancer in both groups were well-balanced (P > 0.05). The detail information was shown in Table 1.

**Table 1:** Comparison of general information between the two groups (n=50)

			ASA classification (n)		Type of pathology (n)		
Group	BMI (kg/m^2^)	Age (years)	Class I	Class II	Invasive ductal carcinoma	Invasive lobular carcinoma	Ductal carcinoma in situ
Single PCIA group	22.89±2.04	52.77±11.02	28 (56.00)	22 (44.00)	29 (58.00)	19 (38.00)	2 (4.00)
Combined group	22.96±2.12	53.96±10.47	31 (62.00)	19 (38.00)	23 (46.00)	24 (48.00)	3 (6.00)
χ^2^/t	0.184	0.606	0.300			1.474	
*P*	0.854	0.545	0.584			0.479	

## Methods

Both groups underwent radical mastectomy under general anesthesia, and anesthesia induction method included intravenous midazolam, propofol, sufentanil, cisatracurium, and the dosage refers to the requirements of the instructions. After the induction, a laryngeal mask was placed and the anesthesia machine was connected. Set the respiratory rate: 12-14 times/min, tidal volume: 6-8 ml/kg, inspiratory/expiratory ratio=1:2, and adjust the partial pressure of carbon dioxide at the end of expiration to 35-45 mmHg. Pump propofol and remifentanil to maintain anesthesia, and the dosage should be referred to the instruction manual for the requirements.

During the operation, cisatracurium was added according to the situation, and the EEG bifrequency index was maintained at 40∼60. The PCIA group was given PCIA analgesic intervention after the operation. Analgesic formula: sufentanil 200 µg, azasetron 10 mg, add saline to 100 mL. background rate 2 mL/h, a single dose of 0.5 mL, lock time 15 min. In the combined group, ultrasound-guided thoracic nerve block combined with PCIA analgesic intervention was given, and the thoracic nerve block was performed after completing the induction of anesthesia. The ultrasound probe was placed below the midclavicular point on the operative side, and the probe was moved laterally toward the anterior axillary line after seeing the axillary static and arteries, and the pectoralis minor and serratus anterior muscles were seen and then punctured in the plane from inside to outside. Ultrasound prompted the tip of the needle into the pectoralis minor and anterior serratus muscle between the injection of 0.375% ropivacaine 3 ml, see the liquid dark area, retracted no gas, blood when prompted by the location of the accurate, continue to inject the 0.375% ropivacaine 20 ml. the tip of the needle retreated to the pectoralis major and pectoralis minor muscles between the injection of 0.375% ropivacaine 10 ml. PCIA analgesia regimen is the same as the control group. The control group chose the PCIA analgesic regimen alone because this method has been widely used and confirmed in the management of postoperative pain in breast cancer, and as a routine method in current clinical practice, its use as a control group can visually demonstrate the relative advantages of ultrasound-guided nerve block combined with PCIA.

### Observation indicators

To assess patients' pain at 3 h, 6 h, 12 h, 24 h and 48 h after surgery, visual analog scoring (VAS) was used^6^, which was divided into resting and active states, and both state scores were 10 out of 10, of which scores higher than 4 required analgesic remedies, and the higher the score, the worse the patient's pain was. This scale's simplicity and sensitivity make it an ideal tool for evaluating the nuanced effects of our analgesic interventions on postoperative pain perception.

Inflammatory markers IL-6, TNF-α, and hs-CRP were selected for their established roles in indicating surgical stress and immune response in breast cancer patients. These markers are pivotal in understanding the inflammatory dynamics post-surgery and evaluating the efficacy of our analgesic approach in mitigating inflammatory responses, crucial for patient recovery and long-term outcomes.

Detection methods: 3 ml venous blood was collected from both groups of patients before and 48 h after operation, and the serum was taken from patients with at least 8h of dietary abstinence. The blood specimen was centrifuged at 4°C for half an hour, and centrifuge speed was set at 3000 r/min for 10 min, and serum was taken from serum for freezing and preservation. The serum was tested for serum interleukin-6 (IL-6), tumor necrosis factor-alpha (TNF-α), high sensitivity-C-reactive protein (hs-CRP), and high sensitivity-C-reactive protein (hs-CRP) using the uniform ELISA method. IL-6, TNF-α and hs-CRP were detected by ELISA. The kits were purchased from Shanghai Zhenke Biotechnology Company Limited, and the enzyme labeling instrument was from Thermo Fisher Scientific, model MK3.

The analgesic effects, postoperative sufentanil consumption, hospitalization conditions, and safety in both groups were recorded.

### Statistical analysis

Statistic Package for Social Science (SPSS) 25.0 (IBM, Armonk, NY, USA) was used for statistical processing, and the measures (including postoperative pain score, effective time of analgesia, postoperative sufentanil dosage, etc.) that were normally distributed (Shapiro-Wilk method test was applied) and variance chi-squared (Levene method test was applied) were described by mean ± standard deviation (χ̅ ± s), and were compared by t-test, and the counting data (including the rate of remedial analgesia, type of pathology, and adverse reactions, etc.) were compared by *χ*^2^ test for comparison, described by applying the number of cases (%), and statistically significant at P<0.05.

## Results

### Comparing postoperative pain differences

**Figure 1:**
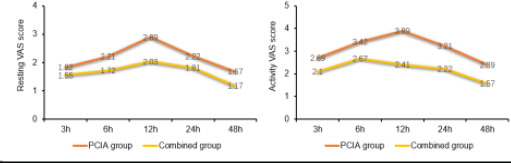
Comparison of postoperative VAS scores between the two groups

The resting and active VAS scores of the combined group at different postoperative times, such as 3h, 6h, 12h, 24h and 48h, were lower than those of the PCIA group at the same time points, which were statistically different (P<0.05). See Table 2 and Figure 1.

**Table 2:** Comparison of postoperative pain differences (n=50, χ̅ ± s)

Score	PCIA group	Combined group	t	*P*
Resting VAS score				
postoperative 3 h	1.82±0.23	1.55±0.21	6.130	0.000
postoperative 6 h	2.21±0.26	1.72±0.25	9.606	0.000
postoperative 12 h	2.89±0.41	2.03±0.34	11.417	0.000
postoperative 24 h	2.22±0.21	1.81±0.18	10.482	0.000
postoperative 48 h	1.67±0.19	1.17±0.16	14.234	0.000
Activity VAS score				
postoperative 3 h	2.69±0.44	2.10±0.31	7.751	0.000
postoperative 6 h	3.42±0.51	2.67±0.46	7.722	0.000
postoperative 12 h	3.89±0.45	2.41±0.39	17.574	0.000
postoperative 24 h	3.21±0.39	2.22±0.32	14.051	0.000
postoperative 48 h	2.39±0.28	1.57±0.21	16.567	0.000

### Comparing differences in inflammatory response

**Figure 2:**
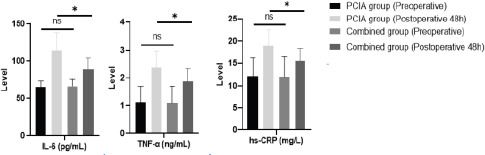
Comparison of inflammatory responses between the two groups of preoperative and postoperative 48h. *P <0.05, ns, no significance

Preoperatively, there was no statistical difference in the comparison of the three inflammatory response factors of IL-6, TNF-α and hs-CRP between the two groups (P>0.05). All three inflammatory responses were elevated in both groups at 48h postoperatively compared with preoperatively, and the three inflammatory responses in the combined group were lower than those in the PCIA group at 48h postoperatively, which was statistically different (P<0.05). See Table 3 and Figure 2.

**Table 3:** Comparison of inflammatory responses between the two groups of preoperative and postoperative 48h (n=50, χ̅ ± s)

	Group	IL-6 (pg/mL)	TNF-α (ng/mL)	hs-CRP (mg/L)		
Preoperative	Postoperative 48 h	Preoperative	Postoperative 48 h	Preoperative	Postoperative 48 h	
PCIA group	65.85±8.41	115.25±22.96	1.12±0.59	2.36±0.61	12.14±4.06	18.98±3.52
Combined group	67.02±9.57	89.57±14.74	1.09±0.63	1.89±0.45	11.97±4.51	15.47±2.97
t	0.649	6.655	0.246	4.384	0.198	5.389
*P*	0.518	0.000	0.806	0.000	0.843	

The postoperative analgesic pump compression, the rate of relief analgesia and the length of hospital stay were compared

**Figure 3:**
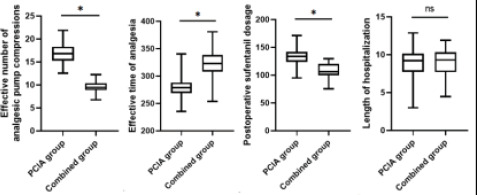
Comparison of effective number of analgesic pump presses, postoperative sufentanil dosage, the effective duration of analgesia and length of hospitalization between the two groups. *P <0.05, ns, no significance

The effective number of analgesic pump presses, postoperative sufentanil dosage and remedial analgesia rate of the combined group were lower than that of the PCIA-only group, and the effective duration of analgesia was higher than that of the PCIA-only group, but there was no statistically significant difference in the length of hospitalization when compared with that of the PCIA group (P>0.05). See Table 4 and Figure 3.

**Table 4:** The postoperative analgesic pump compression, the rate of relief analgesia and the length of hospital stay were compared (n=50)

Group	Effective number of analgesic pump compressions (times)	Effective time of analgesia (min)	Postoperative sufentanil dosage (µg)	Remedial analgesiarate (n)	Length of hospitalization (d)
PCIA group	16.85±2.21	278.85±18.46	133.52±15.89	7 (14.00)	9.25±1.74
Combined group	9.48±1.22	322.58±25.69	108.74±12.65	1 (2.00)	8.97±1.68
t/*χ*^2^	20.644	9.775	8.627	4.891	0.819
*P*	0.000	0.000	0.000	0.027	0.415

### Comparison of the occurrence of postoperative adverse events

In this study, adverse reactions included postoperative pruritus, hypotension, and somnolence, and the monitoring of these reactions was designed to assess the safety of the treatment and to provide important clinical information about the potential risks of combined analgesic approaches. There was no statistical difference in the incidence of adverse reactions such as postoperative itchiness, hypotension, and drowsiness in the combined group compared with the PCIA group (P>0.05). See Table 5.

**Table 5:** Comparison of the occurrence of postoperative adverse reactions (n=50)

Group	Itchiness	Hypotension	Nausea and vomiting	Drowsiness	Dizziness	Total
PCIA group	2 (4.00)	1 (2.00)	1 (2.00)	2 (4.00)	3 (6.00)	9 (18.00)
Combined group	2 (4.00)	2 (4.00)	2 (4.00)	1 (2.00)	1 (2.00)	8 (16.00)
*χ* ^2^						0.069
*P*						0.793

## Discussion

Breast cancer is a malignant tumor caused by uncontrolled reproduction of epithelial cells in the breast, which is most common in women over 40 years old and less common in men^7^. Breast cancer can cause symptoms and signs such as breast lumps, nipple discharge, orange peel changes in the breast skin, and enlarged lymph nodes in the armpits, and it can be diagnosed at an early stage during screening for both cancers^8^. Epidemiological survey found that nearly 300,000 women in China are diagnosed with breast cancer every year, accounting for 24.2% of female malignant tumors^9^. Currently, surgery is generally preferred for breast cancer treatment, whih can remove tumor foci and prolong life. However, the severe pain caused by surgical trauma not only affects the patient's recovery, but also may cause the immune function to be low and lose the surveillance effect on the tumor cells, increasing the risk of postoperative recurrence and metastasis. Therefore, active analgesic treatment should be given after breast cancer surgery^10^,^11^.

PICA is currently a commonly used method of analgesia after breast cancer surgery, which is self-management of pain through patient self-administration of medication by compression^12^. Opioids are the main analgesic used in PICA, which can lead to an increase in the number of opioids used when the patient presses multiple times, resulting in an increase in adverse effects^13^. Combined analgesic mode is a more respected analgesic method in recent years, through the application of other auxiliary analgesic methods to improve the analgesic effect and reduce the amount of opioid analgesic drugs in order to reduce the adverse reactions caused by them. Ultrasound-guided thoracic nerve block is used to block the medial and lateral thoracic nerves by injecting local anesthetic drugs, which is more frequently used in the auxiliary analgesia of thoracic surgery^14^. A study found that the application of thoracic nerve block has a good auxiliary analgesic effect on patients undergoing radical mastectomy for breast cancer^15^.

In this study, we found that VAS scores were lower in the combined group at different times of rest and activity, such as 3 h, 6 h, 12 h, 24 h, and 48 h postoperatively; the number of analgesic pump presses, the dosage of sufentanil in the analgesic pump, and the rate of remedial analgesia were lower, and the effective time of analgesia was longer in the combined group, but the length of hospitalization was similar in the two groups. This result suggests that ultrasound-guided nerve block combined with PCIA analgesia can better reduce postoperative pain and decrease the number of analgesic pump presses, but has little effect on hospitalization time. This is due to the ultrasound-guided nerve block between the pectoralis minor and serratus anterior muscles and between the pectoralis major and pectoralis minor muscles injected with the local anesthetic drug lidocaine, which can produce a reversible block on the nociceptive conduction of nerve fibers, both anesthesia and analgesia, and can play a synergistic analgesic effect when combined with the opioid sufentanil in PICA group, so as to make the patient's pain reduction, reduce the number of analgesic pump presses, the dosage of sufentanil, and the remedial analgesia rate^16^,^17^. The length of hospitalization is affected by a variety of factors such as the patient's incision healing, the presence or absence of infection, and co-morbidities, so the effect of pain and analgesic modalities on the length of hospitalization is not significant.

The body of breast cancer patients in the perioperative period is in a state of oxidative stress, which can cause high expression of hypoxia-inducible factors, induce the expression of pro-inflammatory cytokines IL-6 and TNF-α, and cause inflammatory responses in the body^18^. hs-CRP is a sensitive indicator of inflammation, which can be elevated in the early stage of inflammation^19^. In this study, we found that IL-6, TNF-α, and hs-CRP were elevated in both groups at 48 h postoperatively compared with preoperatively, and IL-6, TNF-α, and hs-CRP in the combined group were lower than those in the PCIA group at 48 h postoperatively. This result suggests that ultrasound-guided nerve block combined with PCIA analgesia inhibits the expression of inflammatory factors. This is due to the fact that ultrasound-guided nerve block combined with PCIA has a better analgesic effect, which reduces the oxidative stress and inflammatory response due to severe pain.

Pruritus, hypotension, nausea and vomiting, drowsiness, and dizziness are common adverse effects of opioids^20^. Although the application of ultrasound-guided nerve block combined with PCIA analgesia can improve the analgesic effect, reduce the number of opioids, and theoretically reduce the risk of adverse reactions. However, this study found that the incidence of adverse reactions such as postoperative skin itching, hypotension, and drowsiness in the combined group was similar to that in the single PCIA group. This result suggests that the addition of ultrasound-guided nerve block to assist analgesia is safer and does not significantly increase the risk of adverse reactions. The sample size of this study was only 50 cases in each group, which may bias the results, and the sample size can be increased in the future to further investigate whether the addition of ultrasound-guided nerve block-assisted analgesia can reduce the risk of adverse reactions.

Although this study demonstrates the potential benefits of ultrasound-guided nerve block combined with PCIA in postoperative pain management in breast cancer, limitations in study design, sample size, and generalizability need to be noted. In particular, the relatively small sample size may affect the broad applicability of the results.

Future studies require larger sample sizes and long-term follow-up to further validate the findings of this study and to explore differences in effectiveness in different populations. In conclusion, ultrasound-guided nerve block combined with PCIA analgesia can reduce the number of effective analgesic pump presses, reduce postoperative pain, and inhibit the expression of inflammatory factors, but it has little effect on the length of hospitalization and adverse effects.
